# Sulforaphane protects intestinal epithelial cells against lipopolysaccharide-induced injury by activating the AMPK/SIRT1/PGC-1ɑ pathway

**DOI:** 10.1080/21655979.2021.1952368

**Published:** 2021-07-24

**Authors:** Yu-jie Zhang, Qian Wu

**Affiliations:** Department of Pharmacy, Xiangyang Central Hospital, Affiliated Hospital of Hubei University of Arts and Science, Xiangyang, China

**Keywords:** Sulforaphane, intestinal epithelial cells, oxidative stress, apoptosis, inflammation, AMPK/SIRT1/PGC-1ɑ pathway

## Abstract

The naturally occurring isothiocyanate sulforaphane, found in vegetables, shows promising anti-inflammatory, anti-apoptosis, and anti-oxidative effects. Whether sulforaphane protects against lipopolysaccharide (LPS)-induced injury in intestinal epithelial cells is unclear. The present study examines the ability of sulforaphane to protect Caco-2 cultures from LPS-induced injury, as well as the mechanism involved. Caco-2 cells were incubated for 24 h with 1 μg/mL LPS and different concentrations of sulforaphane (0.1–10 μM). Then, various indicators of oxidative stress, inflammation, apoptosis, and intestinal permeability were assayed. Sulforaphane increased cell viability and reduced lactate dehydrogenase activity in LPS-treated Caco-2 cells in a concentration-dependent manner. Sulforaphane weakened LPS-induced increases in intestinal epithelial cell permeability and oxidative stress (based on assays of reactive oxygen species, DMA, and H_2_O_2_), and it increased levels of antioxidants (SOD, GPx, CAT and T-AOC). At the same time, sulforaphane weakened the ability of LPS to induce production of inflammatory cytokines (IL-1β, IL-6, IL-8 and TNF-α) and the pro-apoptotic caspases-3 and −9. Sulforaphane also upregulated p-AMPK, SIRT1, and PGC-1ɑ, whose inhibitors antagonized the compound’s protective effects. Sulforaphane can protect intestinal epithelial cells against LPS-induced changes in intestinal permeability, oxidative stress, inflammation, and apoptosis. It appears to act by activating the AMPK/SIRT1/PGC-1ɑ pathway. The drug therefore shows potential for preventing LPS-induced intestinal injury.

## GRAPHICAL ABSTRACT

**Figure:**
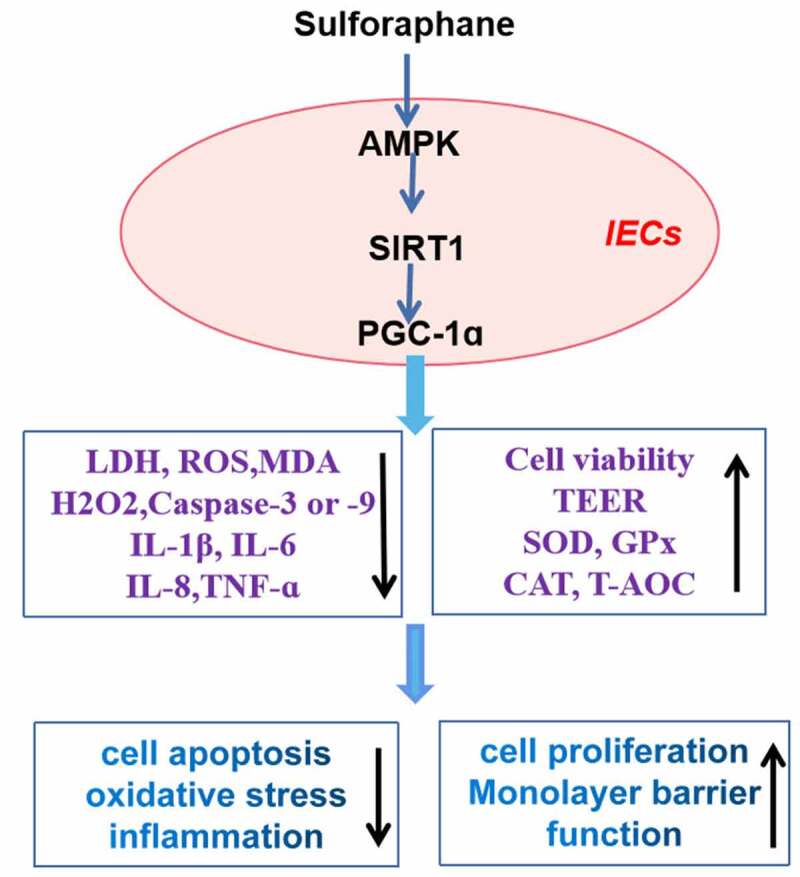

## Introduction

Intestinal epithelial cells (IECs) are an important component of the epithelial barrier, which helps prevent the passage of pathogens, toxins, and allergens from the gastrointestinal lumen into the circulatory system [1,2]. Destruction of the intestinal barrier increases intestinal permeability, destroys homeostasis of the immune system, and induces inflammatory responses and oxidative stress. This can lead to disorders such as inflammatory bowel disease [3], which is characterized by decreased cell proliferation but increased levels of inflammatory cytokines, oxidative stress, and IEC apoptosis [4–6]. Thus, decreasing inflammatory cytokine levels, oxidative stress, and cell apoptosis may help preserve the intestinal epithelial barrier and mitigate inflammatory bowel disease.

In addition to acting as a physical barrier to pathogens, IECs produce mucins, cytokines, and chemokines that prevent harmful microorganisms from invading and colonizing the gut [7]. Pathogen-associated molecular patterns such as lipopolysaccharide (LPS) suppress adenosine monophosphate protein kinase (AMPK) signaling in IECs, which activates Toll-like receptors, resulting in the production of pro-inflammatory cytokines, oxidative stress, and cell apoptosis [8,9]. The signaling cascade mediated by AMPK, silent information regulator 1 (SIRT1), and peroxisome proliferator-activated receptor gamma coactivator 1-alpha (PGC-1α) can inhibit production of reactive oxygen species (ROS) and inflammatory cytokines. Thus, activating the cascade may be an effective therapy against inflammatory bowel disease [10,11].

The isothiocyanate sulforaphane can prevent the progression of several diseases by activating AMPK or SIRT1-mediated signaling transduction [12,13]. The compound exerts anti-inflammatory, anti-oxidative, and anti-apoptotic effects in many tissues [14–16]. Sulforaphane can also protect against IEC injury caused by LPS [17,18], but whether this protection involves the activation of AMPK/SIRT1/PGC-1α signaling is unclear.

The present study examines whether sulforaphane can mitigate LPS-induced IEC damage using cultures of human colonic epithelial cells (Caco-2) as an *in vitro* model. Our experiments also explored whether the effects of sulforaphane on Caco-2 cells involve AMPK/SIRT1/PGC-1α signaling.

## Materials and methods

### Reagents

Sulforaphane, the AMPK inhibitor STO-609, the SIRT1 inhibitor EX527, the PGC-1α inhibitor SR-18,292, and LPS were obtained from Sigma (St. Louis, MO, USA). Fluorescein isothiocyanate-dextran (FITC-D4), 2´,7´‐dichlorofluorescein diacetate (DCFH‐DA), and mitoSox red mitochondrial superoxide indicators were obtained from Gibco (Grand Island, NY, USA). Enzyme-linked immunosorbent assays were purchased from Shanghai Enzyme-linked Biotechnology (Shanghai, China) to determine levels of malondialdehyde (MDA), H_2_O_2_, superoxide dismutase (SOD), glutathione peroxidase (GPx), catalase (CAT), total antioxidative capacity (T-AOC), interleukin-1β (IL-1β), interleukin-6 (IL-6), interleukin-8 (IL-8), and tumor necrosis factor alpha (TNF-α). CCK-8 kit was purchased from eBioscience (Bender MedSystems, Vienna, Austria). Antibodies against AMPK, phospho-AMPK (p-AMPK), SIRT1, or PGC-1α were obtained from Abcam (Cambridge, UK).

### Cell culture and treatment

Caco-2 cells (American Type Culture Collection, Manassas, VA, USA) were maintained in Dulbecco’s modified Eagle’s medium (DMEM) supplemented with 10% fetal bovine serum at 37°C in an atmosphere of 5% CO_2_ in saturated humidity. The medium was changed every 2–3 days. Cells were treated for 24 h with LPS (1 μg/mL) and sulforaphane at final concentrations of 0.1–10 μM. In some experiments, the cells were pre-incubated for 4 h with 2 μM STO-609, EX527, or SR-18,292.

### CCK-8 assay

Caco-2 cells were grown in 96-well culture plates and treated as described in the previous subsection. Then, 10 μL of CCK-8 reagent was added to each well and plates were incubated for 1 h at 37°C under saturated humidity with 5% CO_2_. The optical density (OD) at 450 nm was measured using a microplate reader (Bio-Rad, Hercules, CA, USA). Relative cell viability (%) was defined as OD_experiment/_OD_control_ × 100%.

In parallel, cell viability was assayed in terms of lactate dehydrogenase (LDH) activity. Caco-2 cells were grown in 96-well culture plates, treated as described in the previous subsection; then, LDH in the medium was assayed using LDH Cytotoxicity Assay Kit (Beyotime, Shanghai, China). The OD value at 450 nm was measured using a microplate reader.

### Monolayer barrier function

Monolayer barrier function was assayed as described [19]. Caco-2 cells (2.0 × 10^5^ cells/well) were seeded into a 24-well Transwell^@^ plate (Corning, NY, USA). The medium was changed every day, and when cultures were carried on day 7, the transepithelial electrical resistance (TEER) was nearly 150 Ω•cm^2^. Thus, we continued to culture the cells for another 7 days when the average TEER value was more than 400 Ω•cm^2^. The cultures were then treated with LPS and sulforaphane for 24 h. Next, FITC-D4 was added into the apical chamber and the plate was incubated for 2 h. Medium (50 μL) was recovered from the bottom chamber and added to a plate for fluorescence measurement using an excitation wavelength of 485 nm and emission wavelength of 530 nm.

### ROS

Intracellular ROS levels were assayed using DCFH-DA as described [20]. Briefly, 10 μM of DCFH-DA in DMEM medium was added into each well, and the plate was incubated for 30 min at 37°C in an atmosphere of 5% CO_2_ in saturated humidity. Cells were rinsed three times with phosphate-buffered saline (PBS), then resuspended in PBS. For each sample, the fluorescence intensity of >10^4^ cells was measured at an excitation wavelength of 504 nm and an emission wavelength of 529 nm using a FACSVerse flow cytometer (BD, NY, USA).

Mitochondrial ROS levels were determined using MitoSox red mitochondrial superoxide indicator as described [21]. Briefly, Caco-2 cells were rinsed three times in PBS, then plated into wells containing DMEM supplemented with indicator at a final concentration of 4 mM, and plates were incubated for 20 min at 37°C in an atmosphere of 5% CO_2_ in saturated humidity. Cells were again rinsed in PBS three times, resuspended in PBS, and the fluorescence intensity of >10^4^ cells was measured at an excitation wavelength of 510 nm and emission wavelength of 580 nm using a FACSVerse flow cytometer.

### Oxidative stress and inflammation

After the Caco-2 cells were treated as described in the ‘Cell culture’ subsection, cells were lysed in RIPA buffer (Sigma–Aldrich) on ice for 15 min, then centrifuged at 3.8 × 10^6^ g for 12 min at 4°C, and the supernatant was recovered. Protein concentration in the supernatant was measured using a BCA Protein Assay Kit (Sigma–Aldrich). MDA, H_2_O_2_, SOD, GPx, CAT, and T-AOC were assayed using commercial enzyme-linked immunosorbent assay kits according to the manufacturer’s directions. Similarly, levels of the inflammatory cytokines IL-1β, IL-6, IL-8 and TNF-α were assayed using commercial enzyme-linked immunosorbent assay kits according to the manufacturer’s directions.

### Caspase activity

Supernatants prepared as described in the previous subsection were assayed for activity of caspases-3 and −9 using commercial enzyme-linked immunosorbent assay kits according to the manufacturer’s directions.

### Quantitative reverse transcription-polymerase chain reaction (qRT-PCR)

Total RNA was extracted using Trizol reagent (Sigma–Aldrich). Single-step cDNA synthesis was carried out using the Mir-XTM miRNA First Strand Synthesis Kit (Sigma–Aldrich, St. Louis, MO, USA). Then, 2 μL of cDNA that had been diluted by 1:20 (v/v) was used as template in RT-PCR on a Mx3000 P system (Stratagene, California, USA). The following primers were used:

caspase-3 forward, 5´-ACGCTAAGCTGGGCCCAGTGTTGTACG-3´;

caspase-3 reverse, 5´-GTCAAGCCGGATTTGGCTGAAGCTGAG-3´;

caspase-9 forward, 5´-CCTTGAGTGCATGTAGGCATAATC-3´;

caspase-9 reverse, 5´-CTGGAATGCGTCCTGAAAGTCGATA-3´;

β-actin forward, 5´-GCTTAAGTCGTCCTGATCACTGA-3´;

β-actin reverse, 5´-ACCTGTGTCGTAGCTAGTGCGC-3´.

Transcript levels were expressed relatively to those of β-actin using the 2^−ΔΔCt^ method.

### Western blotting

Caco-2 cells were lysed in RIPA buffer (Sigma–Aldrich) on ice for 15 min. Cell homogenates were centrifuged at 3.8 × 10^6^ g for 12 min at 4°C; then, the supernatant was recovered and quantified for protein using a BCA Protein Assay Kit (Sigma–Aldrich). Proteins (50 g) were fractionated using 10% sodium dodecyl sulfate-polyacrylamide gel electrophoresis and transferred onto nitrocellulose membranes. Nonspecific binding sites on the membrane were blocked with 5% skim milk for 1.5 h at room temperature in a thermostatic incubator. Then blots were then incubated overnight at 4°C with monoclonal antibodies (all diluted 1:1000; Abcam, Cambridge, UK) against the following proteins: AMPK (ab32047), p-AMPK (ab133448), SIRT1 (ab189494), PGC-1ɑ (ab176328), and β-actin (ab8226). Subsequently, blots were washed three times with PBS-Tween 20, then incubated for 2 h at room temperature with horseradish peroxidase-conjugated goat anti-rabbit antibody (diluted 1:4000). Proteins were detected using a luminol reagent and peroxide solution (Millipore, Billerica, MA, USA). Densitometry of images was performed using Image J software.

### Statistical analysis

Data were reported as mean ± standard deviation, and inter-group differences were assessed for significance using an independent-samples *t-*test and one-way analysis of variance using Graphpad 6.0 (Graphpad Prism, Chicago, IL). After performing one-way ANOVA, post hoc tests are required to find statistical differences between groups. Differences associated with *P*< 0.05 were considered significant.

## Results

In our study, we supposed that sulforaphane could improve the inflammatory injury in IECs induced by PLS by activating AMPK/SIRT1/PGC-1ɑ pathway. To confirm the protective effects of sulforaphane against inflammatory damage in LPS-treated cells, we explore its effects on cell proliferation, apoptosis, oxidative stress, inflammatory response, and monolayer barrier function in LPS-exposed IECs. Next, the potential roles of AMPK/SIRT1/PGC-1ɑ axis in IECs were observed.

### Sulforaphane partially reversed LPS-induced death of Caco-2 cells

**Figure 1.**
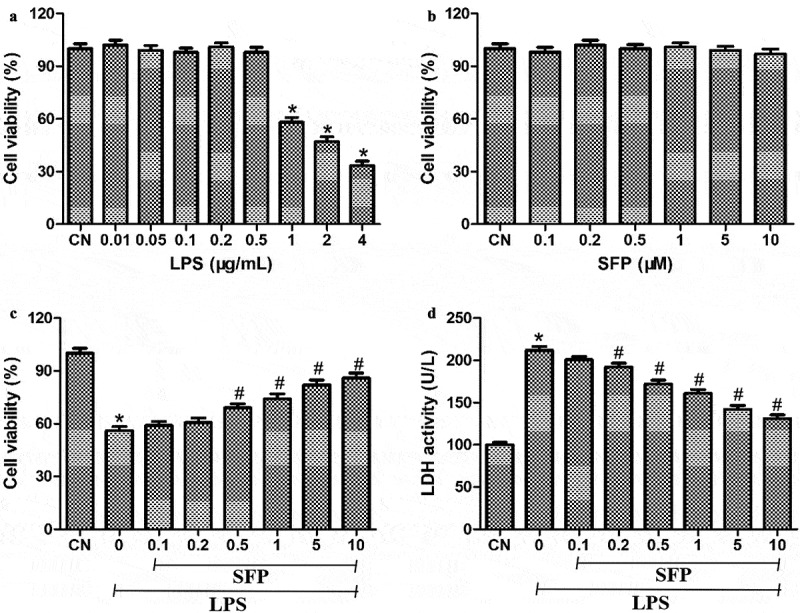
Effects of sulforaphane (SFP) on viability of Caco-2 cells exposed to LPS. (a) Cells were treated with different concentrations of LPS (0.01–4 μg/mL) for 24 h. (b) Cells were treated with different concentrations of sulforaphane (0.1–10 μM) for 24 h. (c) Cells were treated with 1 μg/mL LPS and different concentrations of sulforaphane for 24 h. (d) Cultures treated as in panel (C) were also assayed for LDH activity. Values are mean ± SD (n = 6). Difference between two groups was performed by an independent-samples *t-*test, *P < 0.05 vs. control group (CN); ^#^*P* < 0.05 vs. LPS group. The difference between different concentrations of sulforaphane was performed using one-way analysis of variance

LPS at concentrations higher than 1 μg/mL reduced Caco-2 cell viability by nearly 50%, so 1 μg/mL of LPS was chosen as the concentration for subsequent experiments (Figure 1(a)). No significant cytotoxicity was observed when Caco-2 cells were exposed to sulforaphane at 0.1–10 μM for 24 h (Figure 1(b)). When cultures were exposed for 24 h to 1 μg/mL LPS and sulforaphane at concentrations ranging from 0.1 to 10 μM, viability was significantly higher than in the absence of sulforaphane (Figure 1(c)). Sulforaphane also significantly reversed the LPS-induced increase in LDH activity in Caco-2 cells (Figure 1(d)).

### Sulforaphane mitigated LPS-induced injury of the monolayer barrier function of Caco-2 cells

**Figure 2.**
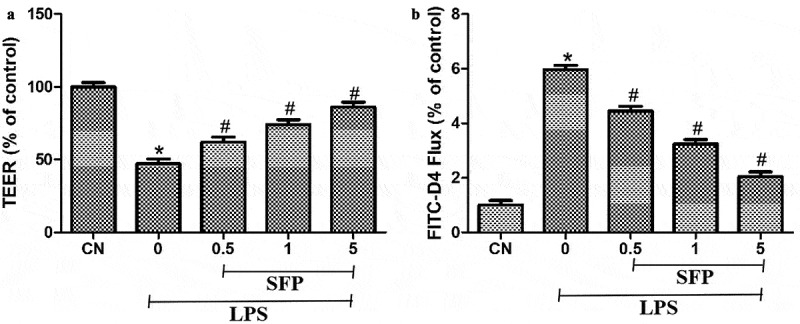
Effects of sulforaphane (SFP) on the monolayer barrier function of Caco-2 cells exposed to LPS. Cells were treated for 24 h with LPS (1 μg/mL) and different concentrations of sulforaphane (0.5–5 μM). (a) TEER measurements. (b) FITC-D4 flux measurements. Values are mean ± SD (n = 6). Difference between two groups was performed by an independent-samples *t-*test, *P < 0.05 vs. control group (CN); ^#^*P* < 0.05 vs. LPS group. The difference between different concentrations of sulforaphane was performed using one-way analysis of variance

To observe the potential protective effects of SFP on monolayer barrier function in intestinal epithelial cells, we measured the TEER and FITC-D4 flux of Caco-2 cells after treatment with LPS and SFP. As expected, LPS significantly reduced the TEER of Caco-2 cells, indicating compromise of the monolayer barrier function (Figure 2(a)). Conversely, LPS significantly increased the FITC-D4 flux of Caco-2 cells (Figure 2(b)). Sulforaphane partially reversed both effects.

### Sulforaphane suppressed LPS-induced oxidative stress in Caco-2 cells

**Figure 3.**
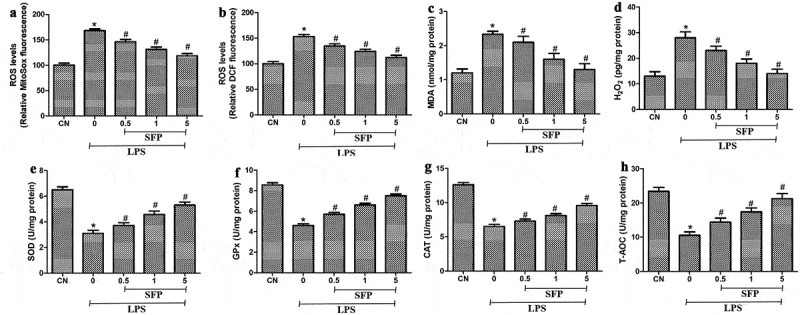
Effects of sulforaphane (SFP) on oxidative stress in Caco-2 cells induced by LPS. Cells were exposed for 24 h to LPS (1 μg/mL) and different concentrations of sulforaphane (0.5–5 μM). (a) Mitochondrial ROS levels, based on MitoSox dye oxidation. (b) Total intracellular ROS levels, based on H2DCF oxidation. (c) MDA levels. (d) H_2_O_2_ levels. (e) SOD activity. (f) GPx levels. (g) CAT activity. (h) T-AOC levels. Values are mean ± SD (n = 6). Difference between two groups was performed by an independent-samples *t-*test, *P < 0.05 vs. control group (CN); ^#^*P* < 0.05 vs. LPS group. The difference between different concentrations of sulforaphane was performed using one-way analysis of variance

We further observe the impacts of SFP on the oxidative stress and antioxidative status in Caco-2 cells via measuring the levels of mitochondrial ROS, intracellular ROS, intracellular MDA, and intracellular H_2_O_2_, and the activities of SOD, GPx, CAT, and T-AOC after exposure to LPS and SFP. LPS's treatment markedly increased the levels of mitochondrial ROS, intracellular ROS, intracellular MDA, and intracellular H_2_O_2_ in Caco-2 cells, while suppressing the levels of SOD, GPx, CAT and T-AOC (Figure 3). These effects were partially reversed by sulforaphane.

### Sulforaphane partially reversed LPS-induced production of inflammatory cytokines in Caco-2 cells

**Figure 4.**
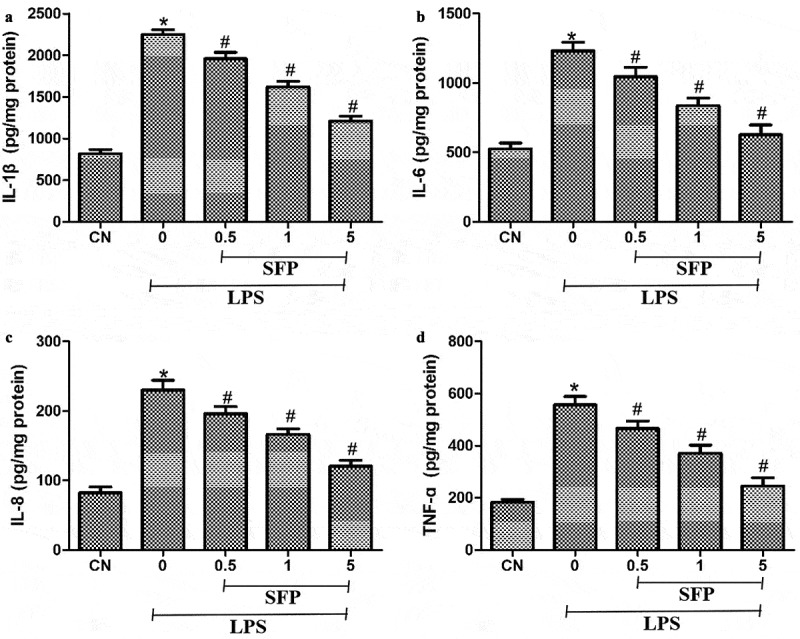
Effects of sulforaphane (SFP) on inflammatory injury in Caco-2 cells induced by LPS. Cells were exposed for 24 h to LPS (1 μg/mL) and different concentrations of sulforaphane (0.5–5 μM). (a) IL-1β. (b) IL-6. (c) IL-8. and (d) TNF-ɑ. Values are mean ± SD (n = 6). Difference between two groups was performed by an independent-samples *t-*test, *P < 0.05 vs. control group (CN); ^#^*P* < 0.05 vs. LPS group. The difference between different concentrations of sulforaphane was performed using one-way analysis of variance

We next investigate the effects of SFP on the inflammatory status of Caco-2 cells via measuring the levels of IL-1β, IL-6, IL-8, and TNF-α after exposure to LPS and SFP. LPS's treatment markedly increased levels of the inflammatory cytokines IL-1β, IL-6, IL-8, and TNF-ɑ (Figure 4). Sulforaphane partially reversed these increases.

### Sulforaphane inhibited LPS-induced apoptosis of Caco-2 cells

**Figure 5.**
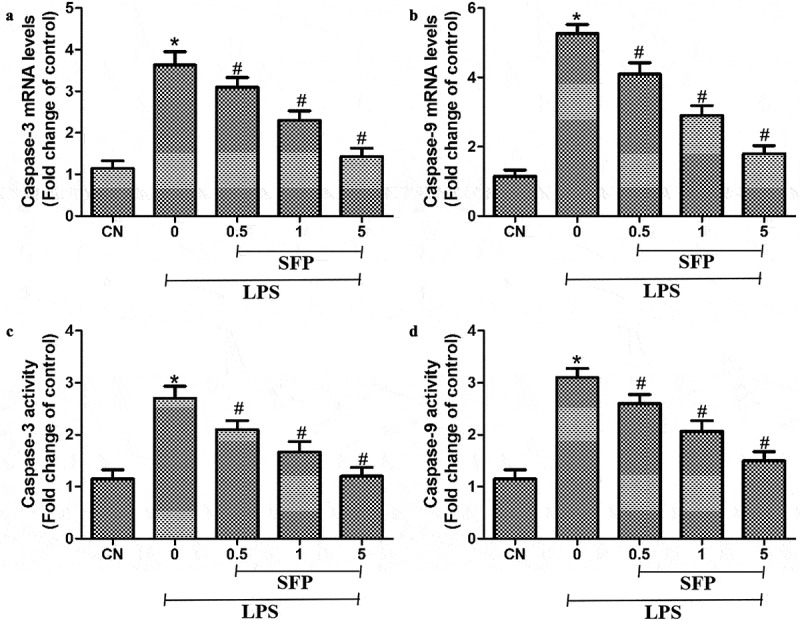
Effects of sulforaphane (SFP) on LPS-induced apoptosis in Caco-2 cells. Cells were exposed for 24 h to LPS (1 μg/mL) and different concentrations of sulforaphane (0.5–5 μM). (a) Levels of caspase-3 mRNA. (b) Levels of caspase-9 mRNA. (c) Activity of caspase-3. (d) Activity of caspase-9. Values are mean ± SD (n = 6). Difference between two groups was performed by an independent-samples *t-*test, *P < 0.05 vs. control group (CN); ^#^*P* < 0.05 vs. LPS group. The difference between different concentrations of sulforaphane was performed using one-way analysis of variance

Additionally, we also explore the protective effects of SFP against the cell apoptosis in Caco-2 cells induced by LPS via detecting the mRNA expression levels and activities of caspase-3 and −9 after exposure to LPS and SFP. LPS markedly increased the levels of the mRNAs encoding caspases-3 and −9, which sulforaphane partially reversed (Figure 5(a,b)). Similar results were observed when cultures were assayed for enzymatic activity of the two caspases (Figure 5(c,d)). We focused on these two enzymes because they help drive apoptosis, such as in LPS-induced intestinal injury.

### Sulforaphane reversed LPS-induced inhibition of the AMPK/SIRT1/PGC-1α pathway

**Figure 6.**
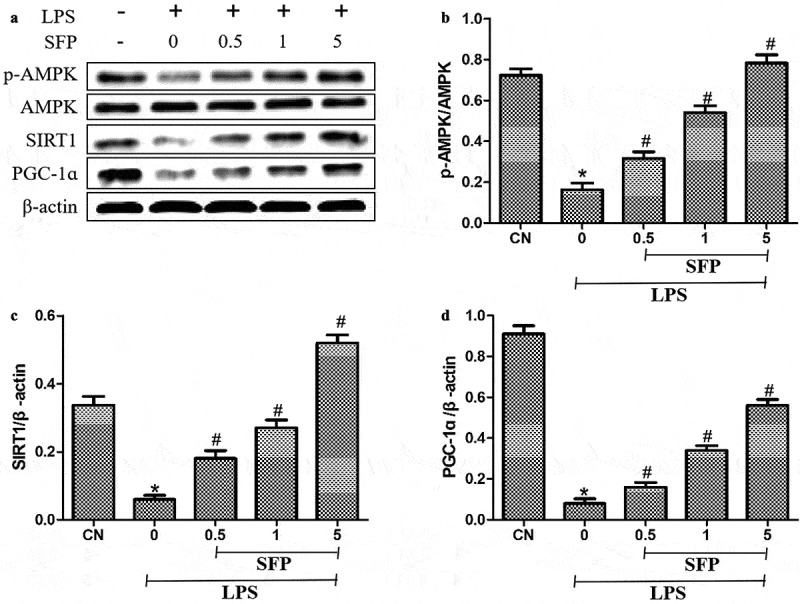
Effects of sulforaphane (SFP) on levels of p-AMPK, SIRT1, and PGC-1ɑ in LPS-treated Caco-2 cells. Cells were exposed for 24 h to LPS (1 μg/mL) and different concentrations of sulforaphane (0.5–5 μM). (a) Representative Western blot. (b-d) Quantitation of Western blots against p-AMPK, SIRT1 and PGC-1ɑ. Values are mean ± SD (n = 3). Difference between two groups was performed by an independent-samples *t-*test, *P < 0.05 vs. control group (CN); ^#^*P* < 0.05 vs. LPS group. The difference between different concentrations of sulforaphane was performed using one-way analysis of variance

To explore how sulforaphane mitigates LPS-induced injury to Caco-2 cells, we focused on the expression of members of the AMPK/SIRT1/PGC-1ɑ pathway. LPS significantly decreased the levels of p-AMPK, SIRT1, and PGC-1α, which sulforaphane was reversed in a dose-dependent manner (Figure 6). These results suggest that sulforaphane may alleviate LPS-induced injury in Caco-2 cells by activating the AMPK/SIRT1/PGC-1α pathway.

### Sulforaphane activated AMPK and the downstream SIRT1/PGC-1α pathway to mitigate LPS-induced injury of Caco-2 cells

**Figure 7.**
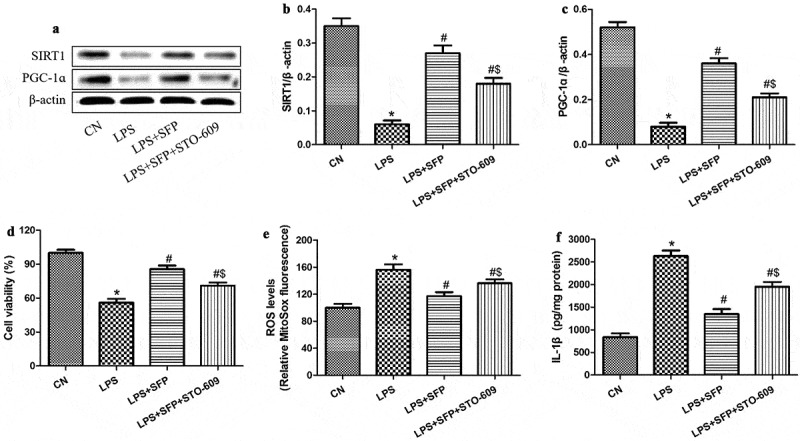
Sulforaphane (SFP) protects Caco-2 cells against LPS-induced injury by activating AMPK. Cells were exposed for 24 h to LPS (1 μg/mL) and SFP (1 μM). (a) Representative Western blot. (b-c) Relative levels of SIRT1 and PGC-1ɑ. (d) Cell viability. (e) Mitochondrial ROS levels, based on MitoSox dye oxidation. (f) IL-1β levels. Values are mean ± SD (n = 3). *P < 0.05 vs. control group (CN); ^#^*P* < 0.05 vs. LPS group; ^$^*P* < 0.05 vs. LPS+SFP group

We exposed the cells to LPS in the presence of sulforaphane and an AMPK inhibitor (STO-609); then, we measured the levels of SIRT1, PGC-1α, ROS, and IL-1β, as well as cell viability. STO-609 partially antagonized the ability of sulforaphane to increase the levels of SIRT1 and PGC-1ɑ (Figure 7(a-c)) and to protect against LPS-induced injury: levels of ROS and IL-1β were significantly higher, and cell viability significantly lower, when AMPK was inhibited (Figure 7(d-f)). These results are consistent with the idea that sulforaphane protects IECs against LPS-induced injury by activating the AMPK cascade.

**Figure 8.**
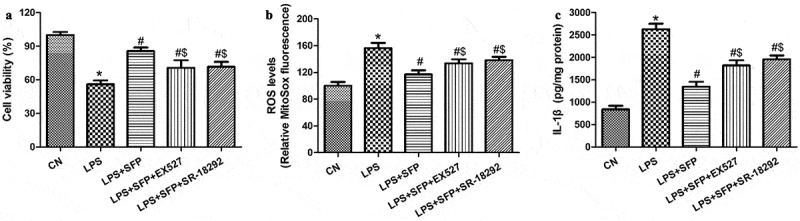
Sulforaphane (SFP) protects Caco-2 cells against LPS-induced injury by activating the AMPK/SIRT1/PGC-1ɑ pathway. Cells were exposed for 24 h to LPS (1 μg/mL) and SFP (1 μM) in the presence or absence of a SIRT1 inhibitor (EX527) or PGC-1ɑ inhibitor (SR-18,292). (a) Cell viability. (b) Mitochondrial ROS levels, based on MitoSox dye oxidation. (c) IL-1β levels. Values are mean ± SD (n = 3). *P < 0.05 vs. control group (CN); ^#^*P* < 0.05 vs. LPS group; ^$^*P* < 0.05 vs. LPS+SFP group

As an additional test of whether sulforaphane exerts its effects by activating the SIRT1/PGC-1α cascade, we exposed cells to LPS and sulforaphane in the presence or absence of a SIRT1 inhibitor (EX527) or PGC-1α inhibitor (SR-18,292), then assayed cell viability and levels of ROS and IL-1β. Either inhibitor antagonized the ability of sulforaphane to promote cell viability (Figure 8(a)) as well as reduce oxidative stress and inflammatory responses (Figure 8(b,c)). These results are consistent with the idea that sulforaphane protects IECs against LPS-induced injury by activating the AMPK/SIRT1/PGC-1ɑ pathway.

## Discussion

Here, we provide evidence that the natural compound sulforaphane can protect against damage to IECs, and that it does so at least partly by activating the AMPK/SIRT1/PGC-1ɑ pathway. We demonstrate that the compound can mitigate the high levels of inflammatory cytokines, oxidative stress, and epithelial permeability induced by LPS, which disrupts the intestinal barrier and thereby contributes to inflammatory bowel disease (IBD) [22,23]. Although the etiology of IBD is still unclear, the interaction between the genetic, environmental or microbial factors, and the immune responses may contribute to its occurrence [5]. Cell apoptosis, necrosis, and autophagy in IECs have been verified to be significantly correlated with the progression of IBD [24]. Additionally, high levels of inflammatory cytokines, oxidative status, and cell permeability have been explored in the patients with IBD [25]. It is well known that the intestine is the most important organ to be involved in metabolism, and its selective permeability maintains that nutrients are absorbed and harmful substances are prevented from entering the body [26]. Symbiotic microorganisms participants in the host metabolize nutrients and protect human health. Nevertheless, harmful microorganisms disrupt host cells via multiple mechanisms, further leading to intestinal and systemic diseases [27]. Gram-negative bacteria, the intestinal microflora, release LPS into the intestinal lumen and further result in dysfunction of IECs [28]. In order to improve IECs functions, we are finding an effective natural compound to treat LPS-induced injury in the intestinal tract. Sulforaphane can also help restore the IEC barrier function [29], as we demonstrated using TEER measurements.

Sulforaphane exerts anti-inflammatory, anti-oxidative, and anti-apoptotic effects, and our assays of inflammatory cytokines, ROS, and caspases suggest that all these effects contribute to the compound’s ability to prevent LPS-induced injury of IECs. These epithelial cells are sensitive to inflammatory processes and to ROS levels that exceed the total antioxidant capacity [30,31]. LPS-induced injury to the intestinal tissue is also associated with increased apoptosis [32]. Consistent with our results, sulforaphane has been shown to protect the gastrointestinal mucosa from oxidative injury and inflammation induced by *H. pylori* and non-steroidal anti-inflammatory drugs [33]. Further experiments should continue to flesh out the details of how sulforaphane protects IECs from injury by reducing inflammation, oxidative stress, and apoptosis.

TEER is a nonspecific marker of the IECs barrier function [34]. Co-cultured with SFP for 24 h, the TEER of Caco-2 monolayer increased strongly, suggesting that SFP could increase the tightness of epithelial cells monolayer. In addition, the results of FITC-D4 test also verify this finding. To explore the underlying mechanism by which SFP decreases intestinal permeability, we also assessed the biological indicators associated with cell viability and apoptosis. Apoptosis is the orderly death of cells controlled by genes (such as caspase-3 and −9) to maintain homeostasis [35]. Early investigation has reported that LPS-induced tissue injury, including intestinal damage, is significantly associated with the increased apoptotic cells. Consistent with this observation, our results also demonstrated that LPS strongly promoted gene expression and enzyme activities of pro-apoptotic molecules (caspase-3 and −9) in Caco-2 cells, whereas SFP reversed these LPS-induced effects. Additionally, the similar results were found in cell viability assay.

There are interconnections in each cellular network to maintain the homeostasis, including AMPK, SIRT1, and PGC-1α. AMPK is a highly conserved serine/threonine protein kinase that helps regulate the levels of ROS in mitochondria [36], and it acts together with its downstream target SIRT1 to upregulate PGC-1α [37] and thereby help control mitochondrial biosynthesis, energy metabolism, and oxidative stress as a homeostasis-sensing network [38]. At the same time, it regulates the activity of SIRT1 and induces the intracellular NAD^+^ which can activate the NAD^+^-dependent SIRT1 to show biological effects. Our results suggest that sulforaphane exerts its effects, at least in part, by activating the AMPK/SIRT1/PGC-1α signaling cascade. This is consistent with reports that sulforaphane alters cellular processes by activating the AMPK signaling [39,40].

## Conclusion

Our experiments suggest that sulforaphane can alleviate LPS-induced IEC damage in the form of increased intestinal permeability, inflammation, oxidative stress, and apoptosis. Furthermore, we showed that the compound exerts these effects by activating the AMPK/SIRT1/PGC-1α cascade. Our data may help guide future studies to develop sulforaphane or suitable derivatives into an effective therapy against inflammatory bowel disease.
